# Assessment of PrEP eligibility and uptake among at-risk MSM participating in a HIV-1 vaccine feasibility cohort in coastal Kenya

**DOI:** 10.12688/wellcomeopenres.15427.2

**Published:** 2020-03-31

**Authors:** Elizabeth Wahome, Susan Graham, Alexander Thiong’o, Oscar Chirro, Khamisi Mohamed, Evans Gichuru, John Mwambi, Matt Price, Eduard J. Sanders

**Affiliations:** 1 KEMRI/Wellcome Trust Research Programme Centre for Geographic Medicine Research– Coast, Kilifi, 80108, Kenya; 2 Department of Medicine, University of Washington, Seattle, Washington, USA; 3 Department of Epidemiology, University of Washington, Seattle, Washington, USA; 4 Department of Global Health, University of Washington, Seattle, Washington, USA; 5 International AIDS Vaccine Initiative, New York, USA; 6 Department of Epidemiology and Biostatistics, University of California, San Francisco, San Francisco, California, USA; 7 Nuffield Department of Medicine, University of Oxford, Headington, UK

**Keywords:** MSM, Receptive anal intercourse, PrEP, Uptake, Risk score, HIV-1

## Abstract

**Introduction:** Pre-exposure prophylaxis (PrEP) is provided free of costs to at-risk populations in Kenya, including men who have sex with men (MSM), but anal intercourse is not an eligibility criterion. We set out to determine PrEP eligibility, uptake and predictors of PrEP uptake among MSM enrolled in an HIV-1 vaccine feasibility cohort in coastal Kenya.

**Methods: **We compared the number of MSM identified as eligible for PrEP from June-December 2017 by Kenyan Ministry of Health (MoH) criteria, which do not include reported anal intercourse, to those identified as eligible by a published MSM cohort-derived HIV-1 risk score (CDHRS). We determined PrEP uptake and assessed factors associated with uptake at first offer among eligible MSM followed up monthly.

**Results: **Out of 167 MSM assessed for PrEP eligibility, 118 (70.7%) were identified by both MoH and CDHRS eligibility criteria; 33 (19.8%) by CDHRS alone, 11 (6.6%) by MoH criteria alone, and 5 (3.0%) by neither criterion. Of the men identified by CDHRS alone, the majority (24 or 72.7%) reported receptive anal intercourse (RAI). Of the 162 MSM eligible for PrEP, 113 (69.7%) accepted PrEP at first offer. Acceptance of PrEP was higher for men reporting RAI (adjusted prevalence ratio [aPR], 1.4; 95% confidence interval [CI], 1.0–1.9), having paid for sex (aPR, 1.3; 95% CI, 1.1–1.6) and group sex (aPR, 1.4; 95% CI, 1.1–1.8), after adjustment for sociodemographic factors.

**Conclusions: **Assessing PrEP eligibility using the CDHRS identified 20% more at-risk MSM for PrEP initiation than when Kenyan MoH criteria were used. Approximately 70% of eligible men accepted PrEP at first offer, suggesting that PrEP is acceptable among at-risk MSM. MSM reporting RAI, group sex, or paying for sex were more likely to accept PrEP. Incorporating RAI into MoH PrEP eligibility criteria would enhance the impact of PrEP programming in Kenya.

## Introduction

Pre-exposure prophylaxis (PrEP) with tenofovir disoproxil fumarate and emtricitabine has been recommended for use in prevention of HIV-1 acquisition ^1^. In response, several countries have implemented country-specific policies and guidelines on PrEP delivery, uptake and monitoring, and a few have made PrEP available nationally to the public ^2, 3^. In Kenya, national PrEP roll-out began in May 2017 after the Ministry of Health (MoH) provide guidance on offering PrEP to HIV-1 negative individuals at substantial ongoing risk of HIV-1 acquisition ^4^. These guidelines, which include several indications to guide PrEP eligibility and initiation among sexually active HIV-1 negative individuals, do not specifically mention receptive anal intercourse (RAI). Despite this gap, men who have sex with men (MSM) are a key population targeted for PrEP ^5, 6^.

MSM have significantly higher risks of HIV-1 acquisition compared to the general population in sub-saharan Africa (SSA) ^7, 8^, and face challenges accessing health care services due to stigma and criminalization of same-sex behavior in these region ^9^. In 2017, we developed an empiric risk score to guide PrEP targeting among at-risk MSM who were followed in a vaccine feasibility study in coastal Kenya with an HIV-1 incidence of 7.0 (95% confidence interval [CI], 5.8-8.6) per 100 person-years. Characteristics of the cohort-derived HIV-1 risk score (CDHRS) included having only male sex partners, RAI, any unprotected sex in the past week, group sex, and young age (18–24 years) ^10^. Because the CDHRS tool demonstrated good performance in predicting HIV-1 acquisition among MSM in this cohort, we were interested in comparing its performance to that of the national MoH guidelines for PrEP eligibility, which were developed for use in all populations at risk for HIV-1 acquisition and not specifically for MSM.

We therefore assessed: 1) performance of the MoH guideline criteria and of the CDHRS to predict HIV-1 acquisition in at-risk MSM using the historic cohort, 2) eligibility for PrEP by either MoH criteria or CDHRS in the cohort since PrEP programming started in June 2017, and 3) PrEP uptake and factors associated with PrEP uptake among MSM cohort participants eligible for PrEP by either criterion. Our ultimate goal is to provide data to optimize MoH guidelines with respect to MSM populations.

## Methods

### Study population

Since July 2005, individuals at risk for HIV-1 acquisition have been recruited for an open cohort study in preparation for a HIV-1 vaccine efficacy trial in a Kenya Medical Research Institute (KEMRI) clinic in Mtwapa town, coastal Kenya. This town, approximately 20 kilometers north of Mombasa, is known for its busy night life and many bars and nightclubs, which are frequented by sex workers ^11^. Participants were identified for recruitment into the study by 10–15 trained peer mobilizers who approached individuals through personal networks and at venues where sex workers meet to establish contact with clients ^12^. Adults aged 18–49 years were eligible if they met any of the following criteria: HIV-1-negative and reporting any of transactional sex work, a sexually transmitted infection (STI) within 6 months, multiple sexual partners, sex with an HIV-1-infected partner, or anal sex during the 3 months before enrolment ^12^. For this analysis, only men who reported anal sex with at least one male partner during follow-up were included.

### Cohort procedures

Detailed cohort procedures have been described elsewhere ^12, 13^. In brief, during enrollment and monthly follow-up visits, a face-to-face interview using a standardized risk behaviour questionnaire, HIV-1 testing and counseling using rapid point of care antibody tests, risk-reduction counselling, medical history and physical examination were performed. During monthly follow-up visits, participants were re-assessed for HIV-1 acquisition risks, treated for genital symptoms suggestive of STIs, offered hepatitis B vaccination and provided with risk reduction counselling. 

***Laboratory evaluation.*** At each study visit, two rapid antibody test kits (Determine, Abbott Laboratories, REF 7D2343; Unigold, Trinity Biotech, REF 1206502) were used in parallel for HIV-1 testing. Discordant rapid HIV-1 test results were resolved using HIV-1 RNA (Xpert® HIV-1 Qual, Cepheid, REF GXHIV-QA-CE-10). Pre- and post-seroconversion samples were tested for HIV-1 RNA using Amplicor Monitor 1.5 (Roche) through 2015, then Xpert® HIV-1 Qual (Cepheid) starting in 2016. Gonococcal infection was diagnosed among participants who reported urethral or rectal symptoms by the detection of Gram-negative, intracellular diplococci consistent with *Neisseria gonorrhoeae* in urethral or rectal secretions ^12^. Prevalent syphilis infection was diagnosed by a positive rapid plasma reagin (RPR, tested annually) titre confirmed by Treponema pallidum haemagglutination assay (TPHA). Incident syphilis was defined as a four-fold increase in RPR titre confirmed by TPHA ^12^.

### Preparing MSM for PrEP uptake

Between January–June 2017, we offered standardized educational messages to all cohort participants who met MoH criteria and to MSM meeting CDHRS criteria but not MoH criteria on the benefits, risks, eligibility and upcoming availability of daily PrEP during individual discussions with clinicians at follow-up visits. In addition, weekly group educational sessions led by counselors were provided to cohort participants who had expressed interest in learning about PrEP. In both individual and group sessions for MSM cohort participants, education included information about known predictors of HIV-1 acquisition among MSM in our cohort, including RAI, group sex, any unprotected sex in the past week, having sex with men only and gonorrhea infection within the past six months ^12^. Because younger age (18–24 years) had become an additional independent predictor in our cohort ^10^, we explained to MSM in this age group their higher risk. Using these identified risk factors in the CDHRS and the MoH PrEP eligibility criteria ^3^, we designed an individualized PrEP eligibility score sheet based on risks reported during the previous three months to target PrEP counseling for MSM cohort participants (see extended data ^14^).

### PrEP rollout

Since June 2017, PrEP has been offered to eligible cohort participants in follow-up. Participants were evaluated for PrEP eligibility at enrollment and monthly follow-up visits during risk reduction counselling sessions by counselors. Those who were eligible by either CDHRS or MoH criteria according to the PrEP Eligibility Score Sheet (see extended data ^14^) were offered PrEP, and their renal function (i.e. creatinine), hepatitis B surface antigen (HBsAg), and symptoms of acute HIV-1 infection (AHI) assessed according to the Kenyan MoH PrEP guidelines ^3^. Participants who tested positive for HBsAg were offered PrEP with close monitoring of liver function while those who tested negative were vaccinated against hepatitis B infection ^13^. Participants who had symptoms compatible with AHI, or those meeting specific risk criteria that increased their risk of HIV-1 acquisition (e.g. RAI, or group sex) were tested for HIV-1 RNA (Xpert® HIV-1 Qual, Cepheid) to rule out AHI prior to PrEP initiation. Participants with no contraindication to PrEP were counselled about the risks, benefits and limitations of PrEP, educated about recognizing AHI symptoms, and provided with a 30-day PrEP supply. Individuals who had previously taken PrEP through other organisations were invited to transfer to KEMRI PrEP programme if they so desired.

During monthly follow-up visits, participants not taking PrEP were reassessed for eligibility and offered PrEP if eligible. Participants taking PrEP completed a computer-assisted self-interview to assess PrEP adherence and motivation to continue PrEP and were monitored for adverse effects, offered syndromic STI treatment as clinically indicated, and tested for HIV-1 (see extended data ^14^). PrEP adherence and sexual risk reduction counseling were provided prior to PrEP refill. Participants with symptoms or signs compatible with AHI were tested for HIV-1 RNA as described above. Participants who tested HIV-1-positive (either on RNA or rapid antibodies) had PrEP discontinued and were counselled and linked to HIV-1 care and treatment.

### Measures

***CDHRS eligibility.*** This variable was defined as having any of the following risk factors at any visit, categorized as either yes or no: age 18–24 years, having only male sex partners, RAI, any unprotected sex (defined as insertive or receptive anal sex or vaginal sex) in the past week, and group sex. Individuals who had any of these risk factors in the 3 months before screening were considered eligible for PrEP by CDHRS criteria.

***MoH eligibility.*** This variable was defined as having any of the following characteristics per MoH PrEP guidelines ^3^ at any visit, categorized as either yes or no: sex with a regular partner of known HIV-1-positive or unknown HIV-1 status in the past week, sex with any partner of known HIV-1-positive or unknown HIV-1 status in the past month, transactional sex (defined as receiving payment for sex with cash, living expenses, or goods) in the past 3 months, sharing needles among people who inject drugs in the past 3 months, sex after alcohol use in the past month, recurrent use of post-exposure prophylaxis (PEP, defined as PEP use more than once in the past 6 months), inconsistent condom use in the past week and STI (defined as a positive gram stain of urethral or rectal secretions or a new syphilis diagnosis within 6 months). Individuals who had any of these characteristics were considered eligible for PrEP by MoH criteria.

Other variables collected at each monthly visit on standardized risk behaviour questionnaire were evaluated as potential predictors of PrEP uptake included the number of reported sexual partners in the past week; paying for sex with cash, living expenses, or goods in the past 3 months; and demographic data collected at enrollment (e.g., education, religion, marital and employment status).

## Data analysis and statistical methods

### Historic cohort before PrEP availability (visits from 2005–2016

***Predicting HIV-1 acquisition.*** We censored data for each participant at the end of 2016, at the last visit (for those lost to follow-up) or at the last seronegative and HIV-1-RNA-negative visit (for those who acquired HIV-1 infection during follow-up). We obtained total observation time for all participants in the study by adding up separate observation times and expressing these in terms of pre-PrEP person-years. To assess the performance of the MoH criteria to identify MSM at risk of HIV-1 acquisition in the historic cohort (2005–2016), we assigned a score of one point to each characteristic (described above) reported and summed these scores to generate a total MoH score for each participant visit. A score of one point was assigned to each characteristic of the CDHRS and a total score was calculated for each visit, following published methodology ^10^. We assessed sensitivity, specificity and area under the receiver operator characteristic (ROC) curve (AUC) for the MoH and CDHRS eligibility criteria using a non-parametric ROC analysis. We compared the AUC for the CDHRS eligibility score to the AUC for the MoH eligibility score using a test of equality of ROC areas.

### Cohort after PrEP availability (visits from June–December 2017

***Data collection.*** Risk behaviour questionnaire, laboratory results, medical history and physical examination data were entered into a secure database. Data were cleaned, recoded and analyzed using Stata 15.0 (StataCorp LP, College Station, TX).

***PrEP eligibility and uptake.*** PrEP provision in limited programmes targeting key populations began in the area around January 2017. Because we did not have reliable data on PrEP use from outside programs, we excluded data collected in the period between January–May 2017. PrEP became available to the KEMRI cohort in June 2017. PrEP baseline was defined as the first study visit by a given participant during June–December 2017. PrEP uptake was defined as acceptance of PrEP by an eligible participant. We ******** censored data for each participant at the end of 2017, at the last visit (for those lost to follow-up) or at the last seronegative and HIV-1-RNA-negative visit (for those who acquired HIV-1 infection during follow-up). Nine MSM who had started PrEP through another program were excluded, as we could not confirm receipt of PrEP. We calculated the number and proportion of MSM eligible by MoH vs. CDHRS criteria at PrEP baseline and presented the results using a Venn diagram. We then compared the proportion of MSM eligible for PrEP by each criterion at baseline and at the last visit in 2017 using McNemar’s test for paired proportions, to determine consistency of PrEP eligibility over time.

We used descriptive statistics to compare baseline demographic and behavioural characteristics of eligible men who accepted PrEP at baseline to eligible men who did not accept PrEP at first offer. We then used generalized linear modeling with log link Poisson regression and robust error variance to identify factors independently associated with PrEP uptake at baseline. Potential predictors of PrEP uptake significant in bivariable analysis at *P* ≤ 0.2 were included in multivariable modeling. *P* values were 2-sided, and significance was set at *P* ≤ 0.05.

### Ethical considerations

The KEMRI Ethics Review Committee approved the study (SSC 894). All participants provided written informed consent.

## Results

### Predicting HIV-1 acquisition

**Figure 1.**
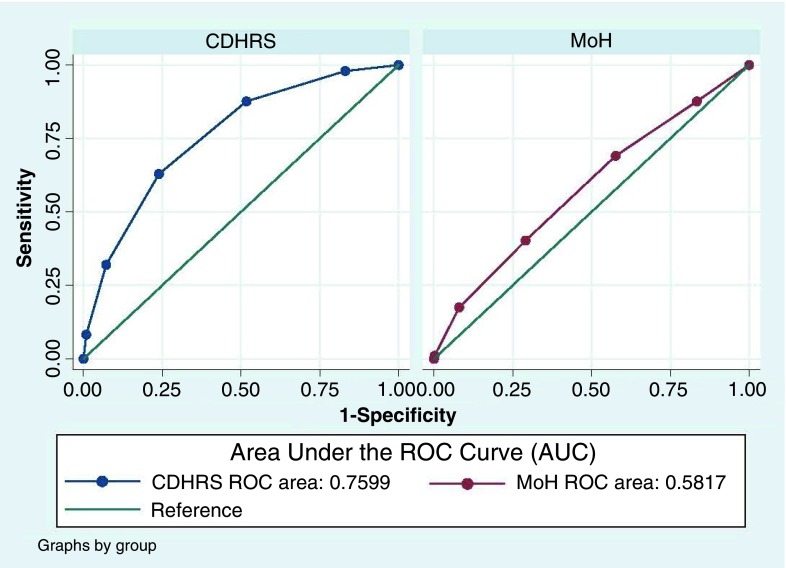
Performance of Ministry of Health (MoH) criteria and cohort-derived HIV-1 risk score (CDHRS). Comparison based on historical data, 2005–2016, Kilifi, Kenya.

From 2005–2016, HIV-1 incidence was 7.0 (95% CI, 5.8-8.6) per 100 person-years. Meeting any of the MoH criteria had a sensitivity of 87.6% and specificity of 16.6%, while meeting any of the CDHRS criteria had a sensitivity of 97.9% and specificity of 16.9% for detecting visits at which men had acquired HIV-1. The AUC for prediction of HIV-1 acquisition for the MoH criteria was 0.58 (95% CI, 0.52-0.64), while the AUC for the CDHRS criteria was 0.76 (95% CI, 0.72-0.80). The comparison between these AUC was significant at *P*< 0.001 ( Figure 1).

### PrEP eligibility

**Figure 2.**
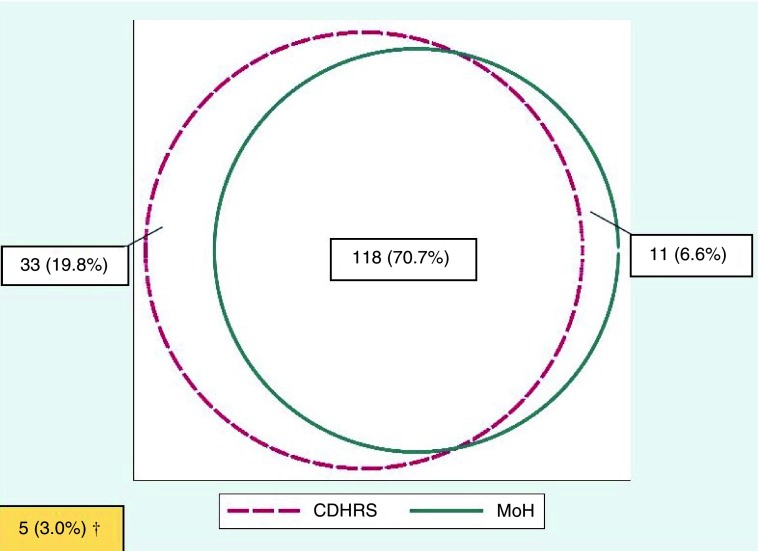
Comparison of Pre-exposure prophylaxis (PrEP) eligibility at baseline among 167 men who have sex with men (MSM), Kilifi, 2017. †Denotes MSM not identified as eligible for PrEP by either the Ministry of Health (MoH) or the cohort-derived HIV-1 risk score (CDHRS) criteria.

Of 167 MSM assessed for PrEP eligibility at baseline in the period June–December 2017, 129 (77.2%) and 151 (90.4%) were eligible for PrEP based on the MoH and the CDHRS criteria, respectively, *P*<0.001. Of these, 118 (70.7%) were eligible for PrEP based on both MoH and CDHRS criteria. However, the CDHRS criteria identified 33 (19.8%) more MSM for PrEP eligibility than the MoH criteria, of whom the majority (24, or 72.7%) reported RAI. In total, 11 (6.6%) men were not identified as eligible by the CDHRS, of whom the majority (6, or 55.0%) reported transactional sex. Five (3.0%) men were not identified as eligible for PrEP by either method, of whom four became eligible for PrEP during follow-up (four based on the MoH criteria and three based on the CDHRS criteria) ( Figure 2). The proportion of MSM eligible for PrEP by either the MoH or CDHRS criteria at baseline and the proportion eligible at their last visit in 2017 were not significantly different, *P* =1.0.

### PrEP uptake

**Figure 3.**
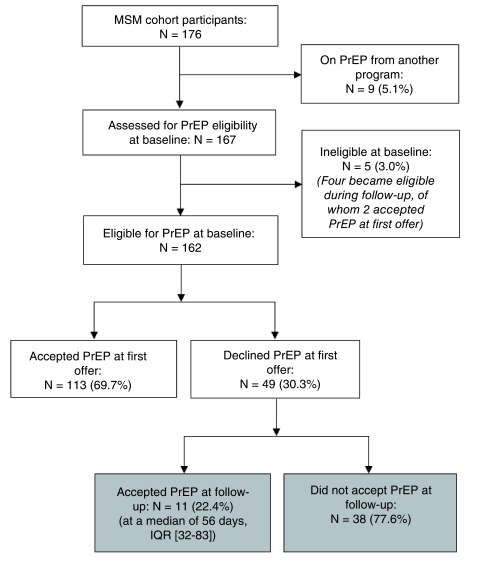
Pre-exposure prophylaxis (PrEP) eligibility and uptake among men who have sex with men (MSM) at baseline, Kilifi, 2017.

Of 162 MSM eligible for PrEP at baseline, 113 (69.7%) accepted PrEP and 49 (30.3%) did not accept PrEP at first offer. Of these 113 who accepted PrEP, 93 (82.3%) and 106 (93.8%) were eligible for PrEP based on the MoH and the CDHRS criteria, respectively, *P*=0.21. Of these 49 who did not accept PrEP at first offer, 11 (22.4%) accepted PrEP during follow-up after a median of 56, interquartile range (IQR) [32–83] days ( Figure 3).

### Baseline of MSM eligible for PrEP

At baseline, of 162 MSM eligible for PrEP, the median age was 26 years, interquartile range (23–30), more than half (57.4%) reported having sex with men only in the past 3 months, one in five (19.8%) had any unprotected sex in the past week, over three-quarters (76.5%) had receptive anal sex and nearly 2% had group sex in the past 3 months (details in Table 1).

**Table 1.** Characteristics of 162 MSM eligible for PrEP at baseline, Kilifi, 2017.

Characteristics	Overall (n = 162)		Declined PrEP at first offer (n = 49)		Accepted PrEP at first offer (n = 113)	
	n (%)		n (%)		n (%)	
Age group (years)						
18–24	66	(40.7)	16	(32.7)	50	(44.2)
25+	96	(59.3)	33	(67.3)	63	(55.8)
Education						
Primary/none	62	(38.3)	20	(40.8)	42	(37.2)
Secondary	84	(51.9)	25	(51.0)	59	(52.2)
Higher/tertiary	16	(9.9)	4	(8.2)	12	(10.6)
Marital status						
Never married	143	(88.3)	40	(81.6)	103	(91.2)
Ever married	19	(11.7)	9	(18.4)	10	(8.8)
Religion						
Christian	88	(54.3)	24	(49.0)	64	(56.6)
Muslim	38	(23.5)	15	(30.6)	23	(20.4)
Other/none	36	(22.2)	10	(20.4)	26	(23.0)
Employment						
None	27	(16.7)	12	(24.5)	15	(13.3)
Self	107	(66.0)	29	(59.2)	78	(69.0)
Formal	28	(17.3)	8	(16.3)	20	(17.7)
Sex of partner in past 3 months						
Men only	93	(57.4)	28	(57.1)	65	(57.5)
Both men and women	66	(40.7)	18	(36.7)	48	(42.5)
Women only	3	(1.9)	3	(6.1)	0	(0.0)
Sexual exposure and protection with condoms in past week						
No activity	64	(39.5)	22	(44.9)	42	(37.2)
All protected	66	(40.7)	20	(40.8)	46	(40.7)
Any unprotected	32	(19.8)	7	(14.3)	25	(22.1)
Number of sex partners in past week						
0	71	(43.8)	23	(46.9)	48	(42.5)
1	35	(21.6)	14	(28.6)	21	(18.6)
2 or more	56	(34.6)	12	(24.5)	44	(38.9)
Receptive anal intercourse (RAI) in past 3 months	124	(76.5)	32	(65.3)	92	(81.4)
Insertive anal intercourse (IAI) past in 3 months	111	(68.5)	32	(65.3)	79	(69.9)
Paid for sex with cash, living expenses, or goods in past 3 months	28	(17.3)	4	(8.2)	24	(21.2)
Received payment for sex with cash, living expenses, or goods in past 3 months	104	(64.2)	27	(55.1)	77	(68.1)
Group sex in past 3 months	3	(1.9)	0	(0.0)	3	(2.7)
Alcohol use in past month	73	(45.1)	23	(46.9)	50	(44.2)
Sex after alcohol use past month	44	(27.2)	16	(32.7)	28	(24.8)
Been raped in past 3 months	1	(0.6)	1	(2.0)	0	(0.0)
People who inject drugs in past 3 months	0	(0.0)	0	(0.0)	0	(0.0)
Recurrent use of post-exposure prophylaxis (PEP) †	3	(1.9)	2	(4.1)	1	(0.9)
Sexually transmitted infection within 6 months ‡	3	(1.9)	1	(2.0)	2	(1.8)
Circumcised	162	(100.0)	49	(100.0)	113	(100.0)

MSM, men who have sex with men; PrEP, Pre-exposure prophylaxis †Defined as post-exposure prophylaxis (PEP) use more than once in the past 6 months. ‡Defined as a positive gram stain of urethral or rectal secretions or a new syphilis diagnosis within 6 months.

In bivariable modeling, PrEP acceptance at first offer was associated at *P*≤0.2 with being younger (18–24 years), never married, self-employed, reporting any unprotected sex in the past week, two or more sexual partners, RAI, having paid for sex, receiving payment for sex and group sex. In multivariable modeling, PrEP uptake was higher for men reporting RAI (adjusted prevalence ratio [aPR], 1.4; 95% CI, 1.0–1.9), men reporting having paid for sex (aPR, 1.3; 95% CI, 1.1–1.6) and men reporting group sex (aPR, 1.4; 95% CI, 1.1–1.8), after adjustment for age, marital status, and employment status. Reporting any unprotected sex in the past week, the number of reported sexual partners, and receiving payment for sex were not associated with PrEP uptake ( Table 2).

**Table 2.** Factors associated with PrEP uptake among 162 MSM eligible for PrEP at baseline.

Characteristics	Bivariable analysis		Multivariable analysis	
	PR (95% CI)	*P* value	aPR (95% CI)	*P* value
Age group (years) †				
18–24	1.2 (0.9-1.4)	0.159	1.1 (0.9-1.4)	0.236
25+	Reference		Reference	
Education				
Primary/none	Reference		-	-
Secondary	1.0 (0.8-1.3)	0.749		
Higher/tertiary	1.1 (0.8-1.5)	0.548		
Marital status †				
Never married	Reference	0.162	Reference	0.216
Ever married	0.7 (0.5-1.1)		0.8 (0.5-1.2)	
Religion				
Christian	Reference		-	-
Muslim	0.8 (0.6-1.0)	0.211		
Other/none	1.0 (0.8-1.3)	0.955		
Employment †				
None	Reference		Reference	
Self	1.3 (0.9-1.9)	0.137	1.3 (0.9-1.9)	0.127
Formal	1.3 (0.9-1.9)	0.232	1.4 (0.9-2.0)	0.126
Sex of partner in past 3 months				
Both men and women	Reference			
Men only	1.0 (0.8-1.2)	0.964	-	-
Sexual exposure and protection with condoms in past week †				
No activity	Reference		Reference	
All protected	1.1 (0.8-1.3)	0.621	1.0 (0.7-1.4)	0.869
Any unprotected	1.2 (0.9-1.5)	0.182	1.2 (0.9-1.7)	0.263
Number of sex partners in past week †				
0	Reference		Reference	
1	0.9 (0.6-1.2)	0.459	0.8 (0.6-1.2)	0.346
2 or more	1.2 (0.9-1.4)	0.164	1.0 (0.8-1.4)	0.780
Receptive anal intercourse (RAI) in past 3 months †				
No	Reference		Reference	
Yes	1.3 (1.0-1.8)	0.059	1.4 (1.0-1.9)	0.039
Insertive anal intercourse (IAI) past in 3 months				
No	Reference		-	-
Yes	1.1 (0.8-1.3)	0.574		
Paid for sex with cash, living expenses, or goods in past 3 months †				
No	Reference		Reference	
Yes	1.3 (1.1-1.6)	0.010	1.3 (1.1-1.6)	0.004
Received payment for sex with cash, living expenses, or goods in past 3 months †				
No	Reference		Reference	
Yes	1.2 (0.9-1.5)	0.136	1.1 (0.8-1.4)	0.543
Group sex in past 3 months †				
No	Reference		Reference	
Yes	1.4 (1.3-1.6)	<0.001	1.4 (1.1-1.8)	0.007
Alcohol use in past month				
No	Reference		-	-
Yes	1.0 (0.8-1.2)	0.754		
Sex after alcohol use past month				
No	Reference		-	-
Yes	0.9 (0.7-1.1)	0.333		
Recurrent use of post-exposure prophylaxis (PEP) ‡				
No	Reference		-	-
Yes	0.5 (0.1-2.4)	0.362		
Sexually transmitted infection within 6 months §				
No	Reference		-	-
Yes	1.0 (0.4-2.1)	0.911		

PR, prevalence ratio; aPR, adjusted prevalence ratio; MSM, men who have sex with men; PrEP, Pre-exposure prophylaxis †Only factors significant at *P* ≤ 0.2 in the bivariable analysis were included in the multivariable model. ‡Defined as post-exposure prophylaxis (PEP) use more than once in the past 6 months. §Defined as a positive gram stain of urethral or rectal secretions or a new syphilis diagnosis within 6 months.

None of the 167 MSM who were offered PrEP acquired HIV-1 in the period June–December 2017.

## Discussion

We showed that among at-risk MSM followed in a historic cohort in the period 2005-2016, MoH criteria for PrEP eligibility were sub-optimal in targeting MSM at risk of HIV-1 acquisition, mainly due to the failure to include RAI as a PrEP eligibility criterion. When programmatic PrEP was offered to eligible MSM cohort participants using the CDHRS, 20% more at-risk MSM were identified for PrEP initiation than when MoH criteria were used. In our setting, 70% of the MSM accepted PrEP at first offer, and uptake was associated with reporting RAI and group sex, suggesting that PrEP is acceptable among MSM at risk of HIV-1 acquisition in Kenya. Of interest is the association of paying for sex with PrEP uptake, as this is not an eligibility criterion included in either the MoH or in our CDHRS tool. While these men met other PrEP eligibility criteria, it could be that paying for sex increases the perception of risk for HIV-1 acquisition among Kenyan MSM.

The fact that the MoH criteria identified MSM at risk for HIV-1 less well among the historic cohort was expected because risk behaviours (e.g., RAI and group sex) ^10, 12, 15, 16^ and sociodemographic factors (i.e., young age) ^10, 17^ known to influence HIV-1 acquisition risk among MSM are not specifically included in Kenyan guidelines. MSM in Kenya and in other parts of SSA have among the highest risks of HIV-1 acquisition compared to the general population ^7, 8^. Previously, MSM reporting RAI have been documented to have a 4–9-fold increased risk of HIV-1 acquisition, independent of other risk factors in Kenya ^10, 12, 15^. Elsewhere, Baggaley *et al.* ^18^ documented the important role played by unprotected anal intercourse in HIV-1 transmission, highlighting the need to include RAI when assessing PrEP eligibility among MSM. The CDHRS tool, on the other hand, omits transactional sex (i.e. receiving payment), which has not been independently associated with HIV-1 acquisition risk in our cohort. The small number of MSM who reported receiving payment for sex but not RAI were therefore captured in the MoH criteria but not in the CDHRS tool.

In the MoH guidelines, healthcare providers are required to assess and discuss HIV-1 acquisition risk without judgment ^3, 19^. As adult male same-sex behaviour is illegal in Kenya and stigma towards MSM is pervasive in health care settings ^20, 21^, many providers may not feel comfortable asking men about same-sex partners or anal sex, leading to missed opportunities for PrEP provision. Moreover, although RAI is also practiced by women, HIV-1 acquisition risk due to RAI in women is underappreciated ^22, 23^. If the Kenyan MoH PrEP guidelines can be updated to include RAI as an indicator for PrEP eligibility, this would help normalize discussions on anal sex and ensure that all individuals at high risk for HIV-1 acquisition are offered PrEP. In addition, sensitization training of health care providers should be facilitated to reduce homophobic attitudes ^24^ and improve MSM healthcare services ^25^.

We report a relatively high PrEP uptake at first offer (70%) among eligible MSM in our study consistent with results documented in other settings in which PrEP uptake ranged between 60% and 93% ^26– 28^ among MSM reporting condomless RAI ^27^ or condomless anal sex ^29^. In addition, we documented higher PrEP uptake among those who reported RAI, group sex or paying for sex. Although RAI and group sex were part of the CDHRS criteria for PrEP eligibility in our cohort ^10^, paying for sex has not been found to be a risk factor for HIV-1 infection in our cohort. In the new MoH ‘HIV self risk checker’ – a rapid tool to assess PrEP eligibility (extended data ^14^)- a question is included on receiving money or favors in exchange for sex: “Have you engaged in: sex in exchange for money or other favors”. Arguably, a person paying for sex may say so, although a question on payment for sex would be preferred. Of note, the question in our risk assessment questionnaire captures payment for sex without specifying whether a male or female partner was paid. Asking men whether they have paid for sex may be one way to identify men who engage in other high-risk sexual behaviors, without pressuring them to admit to male-male sex.

Although we did not find significant differences in PrEP eligibility at baseline compared to the end of the follow-up period in our study, four men became eligible during follow-up, highlighting the importance of period reassessments of HIV risk. We did not encourage men to discontinue PrEP if they no longer met criteria at a follow-up assessment, as we assumed that risk in our cohort would remain substantial. We did not find an association between PrEP uptake and condom use reported in the past week, younger age (18-24 years) or sex of the partner in the past 3 months. While these were eligibility criteria included in our CDHRS tool, it is possible that these risk factors and other risk factors such as partner numbers are less salient in individuals’ self-assessment of HIV-1 risk than RAI, group sex or paying for sex. Qualitative work is needed to explore risk perception in this population and how different behaviors influence PrEP uptake and adherence.

About a third of the eligible MSM participants did not accept PrEP when it was first offered. Of note, 1 in 5 of these men accepted PrEP later in follow-up after the initial refusal. Upon review of their counseling records at baseline, the majority of men who delayed PrEP initiation reported that they were not ready to start. Others opted to continue using condoms, after considering the risks and benefits of PrEP. Further research to understand barriers and facilitators of PrEP uptake among MSM is needed, to target optimal interventions supporting PrEP uptake and adherence among MSM at high risk of HIV-1 acquisition in Kenya ^30^.

Our study had several limitations. First, we did not collect data on partnerships, and so did not establish if any MSM was in a serodiscordant relationship and if so, whether the partner had achieved virologic suppression. Secondly, behavior risk assessment for PrEP initiation was conducted by trained staff with experience in assessing HIV-1 acquisition risks. Therefore, this risk assessment may not reflect risk assessment conducted in non-research settings providing PrEP services in Kenya, and uptake of PrEP by MSM at non-research settings may be lower. Thirdly, in our study, MSM were offered PrEP following a period of counselling on risks and benefits and the importance of PrEP adherence prior to PrEP availability, which may have enhanced uptake once the roll-out occurred. Lastly, our participants were in monthly follow-up, while national programmes recommend 3-montly visits for PrEP maintenance. The intensive counseling provided during monthly visits likely facilitated higher uptake of PrEP among MSM in our cohort.

## Conclusions

Assessing PrEP eligibility in an HIV-1 vaccine feasibility cohort study of MSM at risk of HIV-1 acquisition using a CDHRS identified 20% more at-risk MSM for PrEP initiation than when MoH criteria were used. Most of the additionally identified MSM reported RAI. About 70% of those eligible accepted PrEP at first offer, suggesting PrEP is acceptable among MSM in Kenya. While factors associated with PrEP uptake did not align perfectly with those associated with HIV-1 acquisition risk in this population, RAI was associated with PrEP uptake and should be incorporated into MoH guidelines, to enhance the impact of PrEP programming among MSM and other key populations in Kenya.

## Consent

Written informed consent for publication of the participants details was obtained from the participant.

## Data availability

### Underlying data

Figshare: Dataset assessing PrEP eligibility and uptake among at-risk men who have sex with men (MSM) in Mtwapa, Kenya, https://doi.org/10.6084/m9.figshare.9766613.v1 ^14^

This project contains the following underlying data:

- PrEP Eligibility and Uptake.csv (A spreadsheet consisting of data relating to 986 individuals including demographic information, behaviours, risk factors and uptake of PrEP.)
- Key for Acronyms.xlsx (a key to the acronyms used in column headers in the file PrEP Eligibility and Uptake.csv)

### Extended data

Figshare: Dataset assessing PrEP eligibility and uptake among at-risk men who have sex with men (MSM) in Mtwapa, Kenya, https://doi.org/10.6084/m9.figshare.9766613.v1 ^14^

This project contains the following extended data:

- PSK-Prep-Self-RAST Revised.pdf (one-page HIV Self Risk Checker published by the Kenyan Ministry of Health, including questions on condom use, intravenous drug use, sexually transmitted disease and previous use of PrEP)
- PrEP Adherence 2017-9-4.docx (A blank questionnaire form used to assess the usage of PrEP and participants’ Motivation and Adherence when taking PrEP.)
- PrEP Eligibility Score Sheet.docx (A one-page scoresheet for PrEP eligibility which underpins the Cohort-Derived HIV-1 Risk Score tool (CDHRS) for men who have sex with men.)

Data are available under the terms of the Creative Commons Zero "No rights reserved" data waiver (CC0 1.0 Public domain dedication).
