# Supplementary material for: Assessment of PrEP eligibility and uptake among at-risk MSM participating in a HIV-1 vaccine feasibility cohort in coastal Kenya
Source: Wellcome Open Res. 2020 Mar 31;4:138. Originally published 2019 Sep 19. [Version 2] doi: 10.12688/wellcomeopenres.15427.2 (PMC7043115; doi:10.12688/wellcomeopenres.15427.2)
Supplement: Peer review history

# Peer review: Reviewer response for version 1

**DOI:** 10.21956/wellcomeopenres.16873.r37692

**Published:** 2020-02-25

**Authors:** Amy S. Nunn

**Affiliations:** 1 Department of Behavioral and Social Sciences, Center for Health Equity Research, Brown University School of Public Health, Providence, RI, USA

This is a well designed and well crafted study about acceptability and uptake among MSM in Kenya. Although this is a very high risk group, this population remains understudied in sub-saharan Africa.

 The authors also make important normative arguments about the importance of asking about RAI among men presenting for services. This represents an importance advance to Kenya's response to the epidemic. 

 I have few suggestions to make for this elegant contribution; my only suggestion is to offer a little more information about the MSM epidemic in Sub Saharan Africa and Kenya in the intro and also the discussion.

I confirm that I have read this submission and believe that I have an appropriate level of expertise to confirm that it is of an acceptable scientific standard.

# Response

**Published:** 2020-03-24

**Authors:** Elizabeth Wahome

**Affiliations:** KEMRI-Wellcome Trust Programme, Kenya

**Reviewer #2:**

I have few suggestions to make for this elegant contribution; my only suggestion is to offer a little more information about the MSM epidemic in Sub Saharan Africa and Kenya in the intro and also the discussion. 

*We thank the reviewer for the suggestion. We have included additional information in the introduction about the HIV epidemic among MSM in the sub-Saharan Africa in the introduction (second paragraph) and discussion (second paragraph).*

Finally, we thank you again for your thorough consideration and constructive comments that have helped to improve our manuscript. We trust that the changes made to our revised manuscript will meet your approval.

Yours sincerely,

Elizabeth Wahome, MSc

# Peer review: Reviewer response for version 1

**DOI:** 10.21956/wellcomeopenres.16873.r37019

**Published:** 2019-12-19

**Authors:** Elske Hoornenborg

**Affiliations:** 1 Department of Infectious Diseases, STI Outpatient Clinic, Public Health Service of Amsterdam (GGD), Amsterdam, The Netherlands

Elisabeth Wahome and colleagues have written an interesting and important manuscript about MSM and use of PrEP in Kenya. This key population has special need regarding HIV prevention, and a high uptake of PrEP, being a very effective prevention intervention can have an important impact on the epidemic. They analyzed eligibility criteria according to 2 different scores, and made suggestions for improvement of the currently used criteria. For their analyses, they used data from a well-organized cohort of MSM with a high risk for HIV infection.

 Their research question is clear and relevant, the manuscript is well-written and the analyses are sound. This manuscript can improve implementation of PrEP in several countries.

 I have some suggestions for improvement.

 Abstract: Well written. The numbers in the results section were initially unclear to me, as they omitted that MOH criteria identified an additional number of eligible MSM. I think that this should be added to the abstract.

- The conclusion could be improved for clarification, and stronger position-taking (although this may be a more personal choice): “reinforcing the importance of an informed discussion of HIV-1 risk” to something like MOH criteria should be extended with RAI to better identify MSM at risk for HIV and avoid new HIV infections in this group/ make a larger impact on the epidemic.

Introduction:

- The CDHRS risk score is explained, but the MoH score is not given much detail here, yet. I suggest to introduce both risk scores here, or explain both in the methods section, and emphasize that one was developed for all risk groups, and one specifically for MSM.
- AIM: I could imagine that the aim of this article could also be put as: evaluate whether national guidelines for PrEP could be optimized for MSM populations. That would make it more clear for the reader what the relevance of this article is, and easier to relate to their local epidemic, even if they never heard from the CDHRS before.

Methods:

- Please explain which part of the KEMRI cohort exists of MSM.
- In the “preparing MSM for PrEP” part: explain in the text whether the standardized education etc were only offered to MSM or also to other cohort participants.
- I was a bit confused about the different groups analysed. The cohort participants mentioned in this part, are they part of the KEMRI cohort? Because later on, authors mention that cohort participants are transferred to the KEMRI PrEP cohort. Same for section “PrEP cohort”, which group is this, KEMRI? Please explain a bit more.
- Measures: define “recent”. Does sex include also oral sex?
- Intravenous drug users, better to use people who inject drugs.

Table 1 and Results:

- Why report a p-value here, while a nice uni- and multivariabele comparison is made in Table 2. Consider to leave out the p-value here.
- The authors report that having only female sex partners is associated with declining PrEP, However, as I understand, the p-value reported (0.027) is based on differences between the three categories, men only, both, and women only. This should be adjusted. This also accounts for a few of the other p-values reported in the PrEP uptake section

Discussion:

- Second paragraph: MoH criteria less well identified MSM.
- There is a bit overlap between the 4th and 5th paragraph.
- More elaboration is needed about why some factors are not related with uptake, such as number of partners and use of condoms.

Conclusions:

 From my point of view, you could mention a bit stronger what you would advise, e.g., a risk-assessment tool developed with MSM-specific data identified xx% more persons at risk for HIV compared to the Kenyan national risk assessment tool and should be considered for use to increase the impact of PrEP on the epidemic.

 General point:

 Try to avoid using “ high risk men/MSM”, and rather put: MSM at high risk of HIV, to avoid stigmatization.

I confirm that I have read this submission and believe that I have an appropriate level of expertise to confirm that it is of an acceptable scientific standard.

# Response

**Published:** 2020-03-24

**Authors:** Elizabeth Wahome

**Affiliations:** KEMRI-Wellcome Trust Programme, Kenya

**Reviewer #1:**

Abstract: Well written. The numbers in the results section were initially unclear to me, as they omitted that MOH criteria identified an additional number of eligible MSM. I think that this should be added to the abstract.

*Following the reviewer’s suggestion, we have included the additional number of eligible MSM identified by the MoH criteria as follows: Out of 167 MSM assessed for PrEP eligibility, 118 (70.7%) were identified by both MoH and CDHRS eligibility criteria; 33 (19.8%) by CDHRS alone, 11 (6.6%) by the MoH criteria alone, and 5 (3.0%) by neither criterion.*

The conclusion could be improved for clarification, and stronger position-taking (although this may be a more personal choice): “reinforcing the importance of an informed discussion of HIV-1 risk” to something like MOH criteria should be extended with RAI to better identify MSM at risk for HIV and avoid new HIV infections in this group/ make a larger impact on the epidemic.

*We thank the reviewer for the suggestion. We have changed the conclusion of the abstract to: Incorporating RAI into MoH PrEP eligibility criteria would benefit individuals at risk through this practice and enhance the impact of PrEP programming in Kenya.*

Introduction: The CDHRS risk score is explained, but the MoH score is not given much detail here, yet. I suggest introducing both risk scores here, or explain both in the methods section, and emphasize that one was developed for all risk groups, and one specifically for MSM.

*The following statement has been added in the last sentence of the second paragraph. “Because the CDHRS tool demonstrated good performance in predicting HIV-1 acquisition among MSM in this cohort, we were interested in comparing its performance to that of the national MoH guidelines for PrEP eligibility, which were developed for use in all populations at risk for HIV-1 acquisition and not specifically for MSM.” Additionally, detailed list of the MoH criteria has been included in the fourth paragraph of the methods section under the sub-heading “Preparing MSM for PrEP uptake”.*

**

AIM: I could imagine that the aim of this article could also be put as: evaluate whether national guidelines for PrEP could be optimized for MSM populations. That would make it clearer for the reader what the relevance of this article is, and easier to relate to their local epidemic, even if they never heard from the CDHRS before.

*We thank the reviewer for the suggested aim. We have edited the last sentence of the second paragraph to read “Our ultimate goal is to provide data to optimize MoH guidelines with respect to MSM populations.”*

Methods:

Please explain which part of the KEMRI cohort exists of MSM.

*MSM are included in the KEMRI vaccine feasibility cohort. For this study, we included only data from MSM cohort participants, defined by visits from HIV-1 negative men who reported having anal sex with a man during the 3 months before enrollment. We have clarified further in the last sentence under the sub-heading “Cohort procedures”.*

In the “preparing MSM for PrEP” part: explain in the text whether the standardized education etc were only offered to MSM or also to other cohort participants.

*The standardized educational messages were offered to all cohort participants who met Kenyan MoH criteria, as well as MSM cohort participants who met CDHRS criteria but not MoH criteria.*

**

I was a bit confused about the different groups analysed. The cohort participants mentioned in this part, are they part of the KEMRI cohort? Because later on, authors mention that cohort participants are transferred to the KEMRI PrEP cohort. Same for section “PrEP cohort”, which group is this, KEMRI? Please explain a bit more **.**

*We apologize for this confusion. There is only one cohort, and not a specific “PrEP cohort.” We have clarified the language and now refer to the historic cohort (visits prior to PrEP availability) and the cohort after PrEP availability(visits from June through December 2017).*

**

Measures: define “recent”.

*We apologize for the lack of clarity, and have replaced this word with the specific time frame of reference throughout the document.*

Does sex include also oral sex?

*No, the question on sex with reference to “any unprotected sex” refers to insertive or receptive anal sex or vaginal sex. We have included a definition for sex in the manuscript.*

Intravenous drug users, better to use people who inject drugs.

*We thank the reviewer for the suggestion. We have replaced the term “Intravenous drug users” with “people who inject drugs”*

Table 1 and Results:

Why report a p-value here, while a nice uni- and multivariabele comparison is made in Table 2. Consider leaving out the p-value here.

*Following the reviewer’s suggestion, we have dropped the p-values and revised table 1.*

The authors report that having only female sex partners is associated with declining PrEP, However, as I understand, the p-value reported (0.027) is based on differences between the three categories, men only, both, and women only. This should be adjusted. This also accounts for a few of the other p-values reported in the PrEP uptake section

*The point is well taken. We have revised the paragraph by reporting only the characteristics of all the MSM eligible for PrEP without comparing the two groups, i.e. those who declined vs those who accepted as these associations are already reported in table 2.*

Discussion:

Second paragraph: MoH criteria less well identified MSM.

*We thank the reviewer for the suggestion. The statement has been edited as follows “The fact that the MoH criteria identified MSM at risk for HIV-1 less well…”*

There is a bit overlap between the 4th and 5th paragraph.

*We have revised the 5 ^th^ paragraph by removing the first 3 lines of the paragraph and inserting them in the 2 ^nd^ line of the 4 ^th^ paragraph, as both were referring to PrEP uptake.*

More elaboration is needed about why some factors are not related with uptake, such as number of partners and use of condoms.

*We have added a brief explanation in the 5 ^th^ paragraph as to why we did not find an association between younger men (18-24 years), reporting male sexual partners and any unprotected sex as these were criteria included in our CDHRS. Although there was no association between PrEP uptake and the number of sexual partners in the past week, this criterion was not included in either the MoH or our CDHRS criteria.*

Conclusions:

From my point of view, you could mention a bit stronger what you would advise, e.g., a risk-assessment tool developed with MSM-specific data identified xx% more persons at risk for HIV compared to the Kenyan national risk assessment tool and should be considered for use to increase the impact of PrEP on the epidemic.

*We agree with the reviewer’s point of view; therefore, we have reordered our conclusions paragraph to emphasize consideration of RAI in the MoH guidelines.*

General point:

Try to avoid using “high risk men/MSM”, and rather put: MSM at high risk of HIV, to avoid stigmatization.

*We thank the reviewer for the suggestion. We have replaced the term “high risk men/MSM” with “MSM at risk of HIV-1 acquisition”.*

Finally, we thank you again for your thorough consideration and constructive comments that have helped to improve our manuscript. We trust that the changes made to our revised manuscript will meet your approval.

Yours sincerely,

Elizabeth Wahome, MSc
